# Gamma radiation on seeds of natal grass [*Melinis repens* (Willd.) Zizka] induced plant’s morphological and nutritional variability

**DOI:** 10.1371/journal.pone.0270935

**Published:** 2022-07-29

**Authors:** Raúl Corrales-Lerma, Carlos R. Morales-Nieto, Carlos H. Avendaño-Arrazate, Alan Álvarez-Holguín, Martín Martínez-Salvador, Federico Villarreal-Guerrero

**Affiliations:** 1 Facultad de Zootecnia y Ecología, Universidad Autónoma de Chihuahua, Chihuahua, Chihuahua, México; 2 Instituto Nacional de Investigaciones Forestales, Agrícolas y Pecuarias (INIFAP), Campo Experimental Rosario Izapa, Tuxtla Chico, Tapachula, Chiapas, Mexico; 3 Instituto Nacional de Investigaciones Forestales, Agrícolas y Pecuarias (INIFAP), Campo Experimental La Campana, Aldama, Chihuahua, Mexico; KGUT: Graduate University of Advanced Technology, ISLAMIC REPUBLIC OF IRAN

## Abstract

Induced mutagenesis through gamma radiation generates structural and chemical changes in plants. This study evaluated the morphological and nutritional variability of natal grass [*Melinis repens* (Willd.) Zizka] plants produced from seed irradiated with gamma radiation. Natal grass seed was collected from wild populations in the state of Chihuahua, Mexico. The seed was exposed to a source of Co^60^. The radiation doses were: 0, 10, 50, 100, 150, 200, 250, 300 and 350 Gray (Gy). Sixty-six first generation mutant genotypes (M1), produced from irradiated seed, and nine non-mutant genotypes (M0), developed from non-irradiated seed (0 Gy), were evaluated. For the morphological characterization, 18 variables were measured on the plants when they were at the reproductive stage. The nutritional analysis was performed on the M0, as well as on a group of plants from the M1, which resulted morphologically different (p <0.005) from the rest. The differenced M1 plants were classified as promising mutant genotypes (M1p). Results showed that variability was induced in the M1p. These individuals presented morphological differences in leaf weight-tillering weight ratio and foliage height, compared to the rest of the plants (p <0.001). The M1p 250–10 genotype presented the highest (p <0.001) crude protein and the lowest (p <0.001) lignin contents. Gamma radiation in the seed of natal grass induced morphological and nutritional variability. With that, promising mutant genotypes, with desirable morphological and nutritional attributes, were identified.

## Introduction

Natal grass [*Melinis repens* (Willd.) Zizka] was introduced to the American Continent around 130 years ago [1, 2]. In Mexico, it is dispersed in almost the whole country [3]. This grass has a low protein content, with abundant lignified stems, which decreases its digestibility and preference by livestock and wildlife, compared to other native grasses [4, 5]. Natal grass has been classified as one of the most invasive grasses in the country. That may be due to its ability to get established and to suppress key species native to arid and semi-arid grasslands. In northern Mexico, there is a trend towards the colonization of grasslands by African grasses. For instance, *Eragrostis lehmanniana*, *Cynodon dactylon*, *Cenchrus ciliare* and natal grass are dominant in a large portion of these grasslands [3, 6, 7]. These grasses are already established and controlling their expansion is challenging; hence, they are continuously increasing their surface area. The rusticity of these grasses is a desirable characteristic to revegetate highly degraded soils; however, they are not well accepted by livestock and wildlife due to its low nutritional value. An alternative to control the invasion of introduced grasses may consist in increasing their nutritional value. With that, their consumption may increase, and their consumers may biologically control their invasiveness.

Induced mutagenesis to generate morphological and nutritional variability is a widely used technique in plant breeding of grasses and other crops [8, 9]. The Food and Agriculture Organization of the United Nations and the International Atomic Energy Agency have reported about 3,300 varieties registered as new mutants, obtained from mutagenesis induced with gamma radiation [10]. For instance, in *Sorghum sudanense* and *Brachypodium distachyon*, mutants with high forage value were generated by decreasing the lignin content through gamma radiation [9, 11, 12]. Other grasses such as *Pennisetum purpureum* [13] and *Panicum maximum* [14] have been bred with gamma radiation. Likewise, studies on lawn grasses have shown the induction of genetic variability by mutagenesis. In 1997, the TifEagle variety of bermuda grass was produced. The foliar and tillering morphology was modified in this variety and it is currently used on golf courses due to its resistance to trampling [15]. Other varieties of lawn grasses have been modified by mutagenesis in their size and resistance to water stress [16–18]. *Stenotaphrum secundatum* is another lawn-species in which mutagenesis has been used to generate morphological differences [19]. *Eleusine coracana* and *Pennisetum glaucum* / *typhoides* have been also bred by mutagenesis [8, 11, 20]. These last two species are cultivated in South Asia and Africa to produce grain for human consumption and the production of fodder for livestock.

Nevertheless, the advantages offered by the induced mutagenesis through gamma radiation have not been exploited on the breeding of wild grasses. The rusticity and poor nutritional value reported for natal grass [4, 5], make this wild grass a candidate for plant breeding to obtain improved genotypes for grazing purposes. Studies on inducing variability to natal grass through irradiating its seed with gamma radiation have not been reported before. Hence, this study aimed to evaluate the morphological and nutritional variability in the first generation of natal grass plants produced from gamma-irradiated seeds to identify promising mutants.

## Materials and methods

### Seed collection and irradiation

During October 2014, natal grass seed was collected from 12 wild populations distributed in the state of Chihuahua, Mexico [1]. The seed was mixed all together and then stored during six months. Prior to the irradiation process, a quality analysis was carried out on the seed, which contained 4% of humidity and 35% of the seed germinated [21]. In March 2015, the irradiation process was carried out at the Moscamed Complex of the National Service of Health, Safety and Food Quality (SENASICA) of the Ministry of Agriculture and Rural Development (SADER), located in Metapa de Domínguez, Chiapas, Mexico. Radiation doses were determined using a Gafchromic dosimetry system and a RADCAL ionization chamber, model Accudose (Monrovia, CA. USA). The irradiator was a panoramic Gamma Beam 127 MDS Nordion (Ottawa, ON, Canada) with a 50 g dry Co^60^ storage source. From the stored seed, eight lots of 100 g were extracted. One gram includes between 1300 and 1400 seed approximately [22]. Each lot was irradiated a at different dose. The doses were: 10, 50, 100, 150, 200, 250, 300 and 350 Gray (Gy). In addition, a lot of non-irradiated seed was employed as a control treatment (0 Gy).

### Morphological evaluation

The seeding was performed in March 2015. For that, pots of 25 cm of height with a 10 cm diameter, filled at a height of 21–22 cm with sandy loam soil of alluvial origin, were used. Each germination pot included 20 seed from one irradiation dose. Ten pots from each irradiation dose were employed and the experimental design corresponded to completely randomized blocks. The plants were germinated and grown under greenhouse conditions at the ‘Facultad de Zootecnia y Ecología’, ‘Universidad Autónoma de Chihuahua’, Chih., Mexico. In March 2016, 75 surviving plants from the different irradiation doses were transplanted in black polyethylene pots with dimensions of 30 cm high by 18 cm in diameter. The pots were filled with sandy loam soil of alluvial origin at a height of 25–26 cm. The plants were then pruned 5.0 cm above the tiller crown. During the study, all the plants were homogeneously and periodically watered to brake dormancy and avoid wilting. Plants produced from irradiated seed were tagged as first-generation mutant genotypes (M1) while plants from non-irradiated seed (0 Gy) were tagged as non-mutant genotypes (M0).

For the morphological characterization, the 75 surviving plants were evaluated, with 9, 8, 9, 9, 8, 9, 9, 9 and 5 plants, germinated from seed irradiated at doses of 0, 10, 50, 100, 150, 200, 250, 300 and 350 Gy, respectively. During the reproductive phenological stage, 18 variables were measured on the genotypes based on the varietal guidelines for *Cenchrus ciliare* [23] and *Bouteloua curtipendula* [24]. That was performed to select morphologically differentiated promising mutants (M1p) from M0 genotypes. The variables were: growth habit (°), foliage height (cm), plant height (cm), foliage height-plant height ratio (unitless), tiller diameter (mm), tillering density (#), leaf blade length (cm), leaf blade width (mm), blade length of flag leaf (cm), blade width of flag leaf (mm), chlorophyll concentration index, panicle length (cm), tillering weight (g), leaves weight (g), leaf weight-tillering weight ratio (unitless), seed weight (g), foliage weight without seed (g), and foliage weight-seed weight ratio (unitless). The descriptive statistics resulting from the evaluation of the 18 morphological variables can be consulted in S1 Appendix.

### Nutritional evaluation

To determine the nutritional content, the M1p and M0 genotypes were evaluated in three phenological stages (growth, reproductive, and dormancy). In the growth stage, the foliage was cut between 15 and 23 cm of height after 21 d of regrowth. For the reproductive stage (panicle with immature seed), plants were allowed to grow six weeks after the last cut and then their foliage was pruned. To evaluate the dormancy stage, the sprouts were allowed to grow again and periodic watering was applied during six additional weeks until the plants completed their flowering stage. From that date, watering was gradually reduced to induce dormancy without causing plant’s death. At this stage, which was reached approximately 14 weeks after the previous cut, the aerial biomass was pruned. In all the phenological stages, the cuts on the foliage were made around 5.0 cm above the tiller crown.

The foliage was then dried in a 6M Precision Scientific oven at a constant temperature of 75°C during 48 h. The dry matter was ground to a particle size between 0.1 and 1.0 mm. Fractionation of structural fibers was then carried out with the Ankom’s protocol, based on the method of Van Soest [25] including three samples (repetitions) of dry matter for each M1p plant. For the M0, there were also three repetitions of a bulk sample, which included dry matter from all the genotypes of this group. The crude protein (CP) content was determined separately with the LECO’s protocol based on the DUMAS combustion method [26]. For this test, three repetitions for the M1p and three repetitions for the M0 genotypes were prepared with the same dry matter previously employed for the fiber analysis.

### Monitoring of environmental variables

Inside the greenhouse, temperature and relative humidity of the air (HMP60 and Vaisala Inc., Woburn, MA, USA), as well as solar radiation (LI-200X, Li-Cor, Lincoln, NE, USA) were measured. Data were recorded during the growth and reproductive stages of the plants (June to October 2016) at intervals of one hour in a datalogger (CR1000, Campbell Scientific Inc., Logan, UT, USA). In 2015, the average temperature was 28°C with a maximum of 48°C (June) and a minimum of 14°C (September). The average relative humidity was 26% with a minimum of 15% in June and a maximum of 47% in September. The maximum peak of solar radiation was registered in July with 760 W m^-2^ while the minimum one occurred in August with 650 W m^-2^. For 2016, the temperature mean was 27°C with a maximum of 47°C (June) and a minimum of 13°C (September). The average relative humidity was 28% with a minimum of 17% in June and a maximum of 48% in September.

### Statistical analysis

The data of morphological characterization was analyzed with a Hierarchical Cluster Analysis following the Ward’s linkage method [27]. The grouping criterion was based on the pseudo statistics F, T^2^, and CCC. The Pearson’s coefficient (R^2^) served to determine the level of similarity among groups. In addition, the groups defined in the dendrogram were compared with Multivariate Analysis of Variance with Orthogonal Contrasts. That served to detect multivariate differences among the groups and variables (α = 0.05). The nutritional variables were analyzed separately by phenological stage (i.e. growth, reproductive, and dormancy). In addition, an Analysis of Variance and a multiple comparison of means were performed with the Dunnett’s test (α = 0.05). All the analyzes were carried out with the SAS 9.1.3 software [28].

## Results

### Morphological characterization

**Fig 1:**
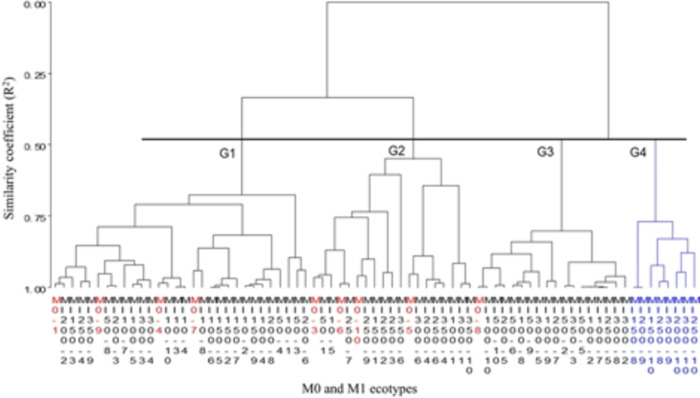
Hierarchical Cluster Analysis with Ward’s linkage, based on 18 morphological variables of 75 genotypes of natal grass [*Melinis repens* (Willd.) Zizka], germinated from seed exposed to different doses of radiation with Co^60^ (M1) and from non-irradiated seed (M0).

The morphological differences among genotypes allowed their grouping by phenotypic affinity (Fig 1).

The absence of M0 (i.e., genotypes from non-irradiated seed) was the criterion to form the groups, based on the pseudo-statistics CCC, F, and T^2^. In total, four groups were formed, which presented a similarity coefficient of R^2^ = 0.48 among groups. The first group (G1) was conformed of 30 individuals including both, M0 and M1 genotypes. This group presented the individuals with the greatest foliage height, plant height, tillering diameter, and leaf length. The second group (G2) integrated 19 genotypes also including both, M0 and M1 genotypes. These genotypes presented the greatest tillering density, leaf width, inflorescence length, leaf weight, tillering weight, and foliage weight. In the third group (G3), 18 genotypes were integrated including only one M0 genotype. This group excluded individuals from seed irradiated at 10 and 150 Gy. The genotypes of G3 presented a straighter growth habit, lower weight of leaves and shorter flag leaves than the rest of the groups. Finally, the fourth group (G4) was conformed of eight M1 genotypes originated from irradiated seed at doses of 150, 200, 250, and 300 Gy. This group was characterized by presenting individuals with lower plant height, shorter inflorescences, thinner tillering, lower tillering density, higher leaves weight-tillering weight ratio, and lower seed weight per plant than the rest of the genotypes. All the groups registered different tillering weight and seed weight (p <0.05).

The inferential analysis (AMOVA) allowed to validate the morphological variability among genotypes. Likewise, it allowed to identify the morphological variables affected by gamma radiation to differentiate promising mutant genotypes. Based on the statistic of Wilks’ Lamda, the four groups formed in Hierarchical Clusters (Fig 1) showed multivariate statistical differences (p <0.001) among themselves. Although G1, G2 and G3 were different from each other, all of them integrated M0 genotypes. Thus, the morphological development of these genotypes was not different from those genotypes coming from non-irradiated seed, regardless of the radiation dose. In this sense, it is difficult to attribute that the phenotypic expression of the nested plants in these three groups was due to an effect of the radiation applied to the seed. Given that, it was necessary to include orthogonal contrasts per group and for each original morphological variable. With this, we determined which variables had an effect to differentiate between one M1 genotype and the M0 genotypes. Such variables were Foliage height and Leaf weight-tillering weight ratio (Table 1). Moreover, it made possible to identify those genotypes presenting desired characteristics to be selected as promising mutants.

**Table 1:** Orthogonal contrasts per group formed in Hierarchical Clusters and for each morphological variable of genotypes of natal grass [*Melinis repens* (Willd.) Zizka], developed from seed irradiated with Co^60^ (M1) and from non-irradiated seed (M0).

Variable	Contrast	F value	P value	Variable	Contrast	F value	P value
Growth habit	G1 vs G2	7.47	0.008	Flag leaf width	G1 vs G2	0.38	0.537
	G1 vs G3	21.54	<0.001		G1 vs G3	1.39	0.243
	G1 vs G4	9.71	0.003		G1 vs G4	1.27	0.264
	G2 vs G3	5.92	0.018		G2 vs G3	0.48	0.493
	G2 vs G4	1.65	0.203		G2 vs G4	0.54	0.464
	G3 vs G4	0.25	0.617		G3 vs G4	0.04	0.838
Foliage height	G1 vs G2	3.45	0.068	Chlorophyll concentration index	G1 vs G2	1.08	0.302
	G1 vs G3	0.07	0.791		G1 vs G3	7.21	0.009
	**G1 vs G4**	**11.05**	**0.001**		G1 vs G4	4.49	0.038
	G2 vs G3	2.35	0.130		G2 vs G3	3.77	0.056
	**G2 vs G4**	**23.91**	**<0.001**		G2 vs G4	2.19	0.144
	**G3 vs G4**	**12.27**	**0.001**		G3 vs G4	0.00	0.982
Plant height	G1 vs G2	28.56	0.001	Inflorescence length	G1 vs G2	0.20	0.653
	G1 vs G3	0.29	0.592		G1 vs G3	14.81	<0.001
	G1 vs G4	23.51	<0.001		G1 vs G4	54.33	<0.001
	G2 vs G3	34.22	<0.001		G2 vs G3	14.45	<0.001
	G2 vs G4	82.33	<0.001		G2 vs G4	55.86	<0.001
	G3 vs G4	19.29	<0.001		G3 vs G4	18.76	<0.001
Foliage height-plant height ratio	G1 vs G2	2.23	0.140	Tiller weight	G1 vs G2	7.70	0.007
	G1 vs G3	0.43	0.514		G1 vs G3	35.46	<0.001
	G1 vs G4	0.00	0.969		G1 vs G4	55.04	<0.001
	G2 vs G3	4.81	0.032		G2 vs G3	14.75	<0.001
	G2 vs G4	1.12	0.293		G2 vs G4	33.80	<0.001
	G3 vs G4	0.30	0.587		G3 vs G4	7.56	0.008
Tillering diameter	G1 vs G2	2.23	0.140	Leaf weight	G1 vs G2	10.06	0.002
	G1 vs G3	0.43	0.514		G1 vs G3	18.04	<0.001
	G1 vs G4	0.00	0.969		G1 vs G4	4.12	0.046
	G2 vs G3	4.81	0.032		G2 vs G3	2.45	0.122
	G2 vs G4	1.12	0.293		G2 vs G4	0.03	0.852
	G3 vs G4	0.30	0.587		G3 vs G4	1.62	0.207
Tiller density	G1 vs G2	59.58	<0.001	Leaf weight-tillering weight ratio	G1 vs G2	0.05	0.819
	G1 vs G3	118.55	<0.001		G1 vs G3	3.26	0.075
	G1 vs G4	174.63	<0.001		**G1 vs G4**	**42.13**	**<0.001**
	G2 vs G3	19.55	<0.001		G2 vs G3	3.91	0.080
	G2 vs G4	69.05	<0.001		**G2 vs G4**	**49.62**	**<0.001**
	G3 vs G4	21.89	<0.001		**G3 vs G4**	**25.42**	**<0.001**
Leaf blade length	G1 vs G2	0.01	0.924	Seed weight	G1 vs G2	14.52	<0.001
	G1 vs G3	0.72	0.400		G1 vs G3	56.92	<0.001
	G1 vs G4	1.48	0.228		G1 vs G4	67.76	<0.001
	G2 vs G3	1.06	0.307		G2 vs G3	20.94	<0.001
	G2 vs G4	1.84	0.179		G2 vs G4	34.94	<0.001
	G3 vs G4	0.30	0.584		G3 vs G4	5.40	0.023
Leaf blade width	G1 vs G2	0.31	0.580	Weight of aerial biomass	G1 vs G2	19.55	<0.001
	G1 vs G3	6.33	0.014		G1 vs G3	65.69	<0.001
	G1 vs G4	9.10	0.004		G1 vs G4	68.22	<0.001
	G2 vs G3	4.97	0.029		G2 vs G3	21.10	<0.001
	G2 vs G4	7.76	0.007		G2 vs G4	30.15	<0.001
	G3 vs G4	1.09	0.299		G3 vs G4	3.68	0.059
Flag leaf length	G1 vs G2	0.63	0.429	Aerial weight biomass- seed weight ratio	G1 vs G2	4.07	0.047
	G1 vs G3	1.09	0.299		G1 vs G3	28.04	<0.001
	G1 vs G4	0.11	0.743		G1 vs G4	59.58	<0.001
	G2 vs G3	0.14	0.712		G2 vs G3	14.88	<0.001
	G2 vs G4	0.06	0.812		G2 vs G4	44.74	<0.001
	G3 vs G4	0.23	0.630		G3 vs G4	12.65	<0.001

P <0.05 indicates statistical difference between two groups per variable, based on Wilks’ Lamda.

For instance, leaf blade length, blade width, and length of the flag leaf were the same for all the groups (p> 0.05). Regarding growth habit, G1, G2, and G3 presented differences (p <0.05) among them. These three groups include both M0 and M1 genotypes. In contrast, G1, G2 and G3 were different (p <0.05) from G4 in growth habit and G4 includes only M1 genotypes. This behavior indicates radiation level in the seed did not generate variability in these variables, since M0 and M1 genotypes were grouped together. Neither foliage height nor leaves weight-tillering weight ratio of the G1, G2, and G3 presented differences (p> 0.05). Nevertheless, these groups had higher foliage height (p <0.05) and lower leaf weight-tillering weight ratio (p <0.05) than G4. This behavior indicates there was an effect of gamma radiation, which induced variability in these two variables for the G4 genotypes.

### Nutritional analysis

Regarding the nutritional content, all the genotypes (M0 and M1p) showed an increase in their content of structural fibers and a decrease in CP throughout their phenological development. Neutral detergent fiber (NDF) in the M1p and M0 ranged between 51 and 61% in the regrowth stage. In the reproductive and dormancy stages, these values ranged from 64 to 74% and from 72 to 75%, respectively. Table 2 shows the hemicellulose, cellulose, lignin, and CP contents in three phenological stages for the different M1p and M0 genotypes. All the M1p presented a difference (p <0.05) with the M0 in at least one nutritional variable. Notably, only the M1p 250–10 genotype showed lower lignin content (p <0.05) than the M0 genotypes in the three phenological stages. Regarding CP, the M1p 250–10 genotype presented higher content (p <0.05) than the M0 genotypes in the three phenological stages.

**Table 2:** Means ± standard error with the Dunnett test for content of structural fibers and crude protein by phenological stage in eight promising mutant genotypes (M1p) of natal grass [*Melinis repens* (Willd.) Zizka], germinated from seed irradiated with Co^60^ vs genotypes germinated from non-irradiated seed (M0).

Stage	M1p y M0	% HEM	% CEL	% LIG	% CP
Growth	M0	30.7±0.63	24.0±0.47	2.8±0.18	17.7±0.23
	M1p 150–8	30.1±0.63	26.6±0.47***	2.2±0.18	16.8±0.23
	M1p 150–10	32.2±0.63	24.2±0.47	2.2±0.18	17.5±0.23
	M1p 200–9	30.9±0.63	25.6±0.47	2.3±0.18	15.9±0.23***
	M1p 200–10	32.6±0.63	22.9±0.47	2.5±0.18	18.7±0.23
	M1p 250–8	31.9±0.63	26.8±0.47***	2.5±0.18	15.3±0.23***
	M1p 250–10	23.9±0.63***	25.4±0.47	1.6±0.18***	19.5±0.23***
	M1p 300–9	30.5±0.63	26.2±0.47	2.3±0.18	15.5±0.23***
	M1p 300–10	32.0±0.63	23.8±0.47	2.4±0.18	17.3±0.23
Reprodutive	M0	35.4±0.5	32.5±0.34	3.8±0.23	11.0±0.11
	M1p 150–8	36.4±0.5	33.2±0.34	3.3±0.23	8.7±0.11***
	M1p 150–10	33.6±0.5	32.4±0.34	3.7±0.23	9.7±0.11***
	M1p 200–9	34.6±0.5	35.9±0.34***	4.3±0.23	8.5±0.11***
	M1p 200–10	34.0±0.5	31.5±0.34	4.1±0.23	9.6±0.11***
	M1p 250–8	33.3±0.5	32.5±0.34	4.3±0.23	10.9±0.11
	M1p 250–10	31.9±0.5***	29.7±0.34	2.3±0.23***	13.6±0.11***
	M1p 300–9	33.3±0.5	32.5±0.34	4.1±0.23	10.7±0.11
	M1p 300–10	33.7±0.5	32.8±0.34	3.9±0.23	8.4±0.11***
Dormancy	M0	34.4±0.46	35.7±0.49	5.2±0.11	6.0±0.14
	M1p 150–8	32.7±0.46	33.8±0.49	5.1±0.11	5.9±0.14
	M1p 150–10	33.3±0.46	35.1±0.49	5.9±0.11***	5.7±0.14
	M1p 200–9	34.3±0.46	34.9±0.49	6.0±0.11***	5.8±0.14
	M1p 200–10	34.4±0.46	35.2±0.49	5.0±0.11	6.0±0.14
	M1p 250–8	33.6±0.46	33.8±0.49	5.0±0.11	5.8±0.14
	M1p 250–10	41.4±0.46***	30.4±0.49***	4.5±0.11***	6.9±0.14***
	M1p 300–9	35.4±0.46	34.0±0.49	5.6±0.11	5.9±0.14
	M1p 300–10	31.9±0.46***	35.5±0.49	5.8±0.11***	6.0±0.14

*** denote differences between M1p and M0 genotypes (p<0.05). HEM = hemicellulose, CEL = cellulose, LIG = lignin, CP = crude protein.

## Discussion

### Morphological characterization

The genotypes belonging to G4 were classified as M1p due to the absence of M0 genotypes in this group. These genotypes were morphologically different to the M0 genotypes and to the rest of the M1 genotypes. Given the observed variability, it was inferred that some morphological mutations, caused by irradiation on the seed, may have occurred in the G4 genotypes.

Even though all the plants were grown under similar conditions, different phenotypic expressions were observed on them. Such expressions did not depend on the radiation level given to the seed, since all the groups included M1 ecotypes from different radiation doses. Gamma rays can induce changes in the genome structure; however, they occur randomly and will largely depend on the radiation intensity. Thus, when the mutagenesis is induced by physical agents such as Co^60^, it is necessary to determine optimal doses for more effective mutations [21, 29–31].

The grouping of the genotypes, presented by the cluster analysis, allowed to infer that gamma radiation induced morphological changes in some of the genotypes, since all the groups registered different tillering weight and seed weight. Previous research has reported intraspecific variation after the induction of mutations, which may possibly generate either new attributes or present some of those the species has lost during its evolution process [32, 33]. Results from the Cluster and the AMOVA analyses allowed to infer that the phenotypic variability found in the M1p genotypes was caused by mutagenesis induced with gamma radiation, which confirms the effect of radiation on expression.

### Nutritional analysis

The nutritional value found in this study is common for grasses, where CP is decreasing and structural carbohydrates such as hemicellulose, cellulose and lignin are increasing along the plant’s development [34–36].

The total of structural fibers of natal grass found in this study was high, compared to the results presented by Bezabih et al. [37], who reported from 54.2 to 56.4% of NDF for this species, in semi-arid grasslands of the savanna of the Rift Valley in Ethiopia during August. The difference in structural fibers between the plants grown in Chihuahua and those grown in South Africa was around 22% for the same phenological stage, which can be considered high. This may be due to the differences in climate conditions, soil nutrients, genetic variability, among other factors.

For ruminants, cellulose is more digestible than hemicellulose while hemicellulose is more digestible than cellulose for non-ruminants. For both of them, lignin is indigestible [38, 39]. Other authors have reported that natal grass with a high lignin content [5]; however, they did not report amounts for this polymer.

In general, the ecotype M1p 250–10 was superior to those reported by other researchers for natal grass. For instance, Melgoza et al. [5] reported values from 4.0 to 6.0% of CP during the growth stage. Ramírez et al. [40] reported 7.4, 11.2, 11.1, and 9.3% of CP content; 27, 29, 28, and 28% of cellulose, and 6, 8, 8, and 8% of lignin, during the spring, summer, autumn, and winter seasons, respectively. González-García et al. [41] reported 3.58% of CP, 3.53% of lignin, 42.89% of cellulose, and 26.29% of hemicellulose in August, when the species began its flowering stage in the grasslands of northwest Chihuahua. Njau et al. [42] reported 3.21% of CP in the dry season in Tanzania. Souza et al. [43] found 1.4% of total nitrogen, which corresponds to 8.75% of CP, applying the correction factor of 6.25; however, they did not specify the plant phenological stage. In a study of the nutritional value of cattle diet in a rangeland located in Chihuahua, which was mainly composed of natal grass (87%), Gutiérrez et al. [44] found 13.2, 10.7, and 6.5% of CP in the growth, flowering, and dormancy stages, respectively. These authors determined values of 70, 71, and 72% of NDF in the growth, flowering, and dormancy stages, respectively. These latter are the closest compared to the values found in the present study. Thus, all the natal grass populations in the state of Chihuahua may have a similar nutritional content.

Given the nutritional content of natal grass found in this study, it could be questioned to postulate that populations of this species in Chihuahua are of poor forage value. This invasive species showed greater CP values in the growth and flowering stage than native species qualified to have a good nutritional value. For instance, Morales et al. [45] reported that *Bouteloua gracilis* contains 12 to 15% of CP in the growth stage in Chihuahua. In *Bouteloua gracilis* var. Cecilia, Beltrán et al. [46] reported 9.7% CP and 3.4% in flowering and maturity stages, respectively. For *Bouteloua curtipendula* var. Diana, 8.6% and 3.6% of CP were reported in the flowering and maturity stages, respectively [47]. However, CP is not the only considered variable to qualify a forage source as good. For that, it would be necessary to determine digestibility and acceptance by cattle and wildlife, among other attributes.

Although all the M1p genotypes were nutritionally different from the M0 genotypes, most of them presented a lower nutritional value. The exception was the M1p 250–10 genotype, which presented the highest CP and the lowest lignin contents in all the phenological stages evaluated. This same ecotype decreased the content of cellulose, hemicellulose and lignin structural fibers in its growth and reproductive stages. During the dormancy stage, it reduced its cellulose and increased its hemicellulose contents. It should be noted that protein is the scarcest nutrient during the dry season and the one most searched by cattle and wildlife. The low preference cattle show for some species of poor nutritional value may be due to the high tillering density or the low leaf-tillering ratio these species generally have. These attributes are correlated to a high content of structural fibers and to a low PC content [41, 48]. In general, the M1p 250–10 genotype can be considered as a promising mutant, since the first generation this genotype surpassed the M0 and M1 genotypes in nutritional value in all the phenological stages.

Although gamma radiation from Co^60^ randomly induces mutagenesis, it was observed that all the M1p genotypes came from irradiated seed at doses ranging from 150 and 300 Gy. Thus, the probability of achieving effective mutations and then promising mutants could be increased when an optimal radiation dose is determined previously. In this regard, some researchers reported that effective mutations occur around the optimal dose estimated for the species to be mutated [49, 50]. In a study conducted by Corrales-Lerma et al. [21], the optimal radiation dose in natal grass seed for effective mutations was determined to be around 300 Gy. The M1p 250–10 genotype was produced from a seed irradiated at 250 Gy, a value close to the optimal radiation dose previously found for natal grass seed. The results from the nutritional analysis and morphological variability validate the effect of gamma radiation applied to the seed, which produced the M1p 250–10 genotype.

## Conclusions

Seeds of natal grass irradiated with gamma radiation produced plants with morphological and nutritional variability. From these plants, it was possible to identify promising mutant genotypes. The genotype identified as M1p 250–10 presented desirable morphological and nutritional attributes of agronomic interest such as, foliage height, leaf-tillering ratio, as well as a reduction in lignin content and an increase in crude protein, compared to the genotypes produced from non-irradiated seed.

## Supporting information

S1 Appendix (DOCX)

## References

[1] Corrales-Lerma R, Morales-Nieto CR, Villarreal-Guerrero F, Santellano-Estrada E, Melgoza-Castillo A, Álvarez-Holguín, et al. Caracterización morfológica y nutricional de pasto rosado [*Melinis repens* (willd.) zizka] en el estado de Chihuahua. *Agroproductividad*. 2017; 10: 103–109. https://www.revista-agroproductividad.org/index.php/agroproductividad/article/view/77

[2] Stokes CA, MacDonald GE, Adams CR, Langeland KA, Miller DL. Seed biology and ecology of natal grass (*Melinis repens*). Weed Science. 2011; 59: 527–532. doi: 10.1614/WS-D-11-00028.1

[3] Herrera-Arrieta Y, Cortés-Ortiz A. Diversidad de las gramíneas de Durango, México. *Polibotánica*. 2009; 28: 49–68.

[4] Terán-Romo A. Índice de consumo de especies *Bouteloua gracilis* y *Melinis repens* y su efecto en la composición fisicoquímica del suelo. Tesis de Maestría. Instituto Politécnico Nacional. 2010. Available from: https://www.semanticscholar.org/paper/%C3%8Dndice-de-consumo-de-especies-Bouteloua-gracilis-y-Durango/f943e0da48e46b409d9638c1902a5eb2a4ce567a

[5] Melgoza CA, Valladares MI, Mata R, Pinedo C. Biología del pasto rosado *Melinis repens* e implicaciones para su aprovechamiento o control. Revisión. Revista Mexicana de Ciencias Pecuarias. 2014; 5:429–442.

[6] Díaz A, Flores E, De Luna A, Luna JJ, Frías JT, Olalde V. Biomasa aérea, cantidad y calidad de semilla de *Melinis repens* (Willd.) Zizka, en Aguascalientes, México. Revista Mexicana de Ciencias Pecuarias. 2012; 3:33–47.

[7] Pinedo AC, Hernández-Quiroz NS, Melgoza A, Rentería VM, Vélez SVC, Morales NCR, et al. Diagnóstico Actual y Sustentabilidad de los Pastizales del estado de Chihuahua ante el Cambio Climático. 2013. Available from: http://10.0.0.98/xmlui/handle/1/1565

[8] Ambavane AR, Sawardekar SV, Sawantdesai SA, Gokhale NB. Studies on mutagenic effectiveness and efficiency of gamma rays and its effect on quantitative traits in finger millet (*Eleusine coracana* L. Gaertn). Journal of Radiation Research and Applied Sciences. 2015; 8: 120–125. doi: 10.1016/j.jrras.2014.12.004

[9] Golubinova I, Naydenova Y, Enchev S, Kikindonov T, Ilieva A, Marinov-Serafimov P. Biochemical evaluation of forage quality from mutant forms Sudan grass (*Sorghum sudanense* (Piper) Stapf.). Ecology and Future—Journal of Ecology and Environment Sciences (In Bulgarian, with English abstract). 2016; 15: 43–51.

[10] MVD—Mutant Variety Database. [Cited 2021 March 27]. Available from: http://mvd.iaea.org/

[11] Ousmane SD, Elegba W, Danso K. Radio-sensibility of pearl millet [*Pennisetum glaucum* (L.) R. Br] and cowpea [*Vigna unguiculata* (L.) Walp] seeds germination and seedling growth. International Journal of Innovation and Applied Studies. 2013; 4:665–671.

[12] Lee MB, Kim JY, Seo YW. Identification of lignin‐deficient *Brachypodium distachyon* (L.) Beauv. mutants induced by gamma radiation. Journal of the Science of Food and Agriculture. 2017; 97; 2159–2165. doi: 10.1002/jsfa.8024 27604502

[13] Pongtongkam P, Nilratnisakorn S, Piyachoknakul S, Thongpan A, Aranananth J, Kowitwanich K, et al. Inducing salt tolerance in purple guinea grass (*Panicum maximum* TD58) via gamma irradiation and tissue culture. Natural Sciences. 2005; 39: 681–688.

[14] Lajonchere G, Mesa AR, Prieto M, Sánchez E. Curva de radiosensibilidad con ^60^Co en guinea (*Panicum maximum* Jacq.) cv. K-249. Revista Pastos y Forrajes. 1995; 1: 35–42.

[15] Hanna W, Elsner E. Registration of “TifEagle” bermudagrass. Crop Science. 1999; 39: 1258.

[16] Lu S, Wang Z, Niu Y, Guo Z. Antioxidant responses of radiation-induced dwarf mutants of bermudagrass to drought stress. Journal of the American Society for Horticultural Science. 2008; 133: 360–366. doi: 10.21273/JASHS.133.3.360

[17] Chen C, Lu S, Chen Y, Wang Z, Niu Y, Gu Z. A gamma-ray–induced dwarf mutant from seeded bermudagrass and its physiological responses to drought stress. Journal of the American Society for Horticultural Science. 2009; 134: 22–30. doi: 10.21273/JASHS.134.1.22

[18] Lu S, Wang Z, Niu Y, Chen Y, Chen H, Fan Z, et al. Gamma-ray radiation induced dwarf mutants of turf-type bermudagrass. *Plant Breeding*. 2009; 128: 205–209. doi: 10.1111/j.1439-0523.2008.01544.x

[19] Li R, Bruneau AH, Qu R. Morphological mutants of St. Augustine grass induced by gamma ray irradiation. Plant Breeding. 2010; 129: 412–416. doi: 10.1111/j.1439-0523.2009.01735.x

[20] Ambli K, Mullainathan L. Effect of gamma rays and ems on seed germination and seed characters in pearl millet (*Pennisetum typhoides*) (Burn.) Stapf. Var. CO (Cu)-9. Journal of Chemical, Biological and Physical Sciences. 2014; 4: 3345–3349. http://www.jcbsc.org/test/issuebio.php?volume=4&issue=4

[21] Corrales-Lerma R, Avendaño-Arrazate CH, Morales-Nieto CR, Santellano-Estrada E, Villarreal-Guerrero F, Melgoza-Castillo A, et al. Radiación gamma para inducción de mutagénesis en pasto rosado [*Melinis repens* (Willd.) Zizka]. Acta Universitaria. 2019; 29: 1–10. doi: 10.15174/au.2019.1847

[22] Carrillo-Saucedo SM, Arredondo-Moreno T, Huber-Sannwalda E, Flores-Rivas J. Comparación en la germinación de semillas y crecimiento de plántulas entre gramíneas nativas y exóticas del pastizal semiárido. Técnica Pecuaria en México. 2009; 47:299–312. https://www.redalyc.org/articulo.oa?id=61312111006

[23] Guiot GJD, Salomé S, Quero AR, Carballo A, Enríquez J, Beltrán S. Guía técnica para la descripción varietal de pasto buffel (*Cenchrus ciliaris* L.). *Guía Técnica Varietal*. SNICS-SAGARPA, México. 2014: 22 p. doi: 10.13140/2.1.2787.3604

[24] Quero CAR, Guiot JD, Salinas S, Enríquez JF, Beltrán S, Tovar, et al. Guía técnica para la descripción varietal de pasto banderita [*Bouteloua curtipendula* (Michx.) Torr.]. Guía Técnica Varietal. SNICS-SAGARPA. México. 2014; 23 p. doi: 10.13140/2.1.3573.7921

[25] Van Soest PJ. Use of detergents in the analysis of fibrous feeds. ii. a rapid method for the determination of fiber and lignin. Journal of Association of Official Analytical Chemists. 1963; 46: 829–835. doi: 10.1093/jaoac/73.4.491

[26] Watson ME, Galliher TL. Comparison of Dumas and Kjeldahl methods with automatic analyzers on agricultural samples under routine rapid analysis conditions. Communications in Soil Science and Plant Analysis. 2001; 32; 2007–2019. doi: 10.1081/CSS-120000265

[27] Núñez-Colín CA, Escobedo-López D. Uso correcto del Análisis Clúster en la caracterización de germoplasma vegetal. Agronomía Mesoamericana. 2011; 22: 415–427. doi: 10.15517/AM.V22I2.8746

[28] SAS, Institute Inc. Statistical Analysis Sytillering 9.1.3 User´s guide. 2006. Cary, NC, USA.

[29] Songsri P, Suriharn B, Sanitchon J, Srisawangwong S, Kesmala T. Effects of Gamma radiation on germination and growth characteristics of physic nut (*Jatropha curcas* L.). Journal of Biological Sciences. 2011; 11: 268–274. doi: 10.3923/jbs.2011.268.274

[30] Thole V, Peraldi A, Worland B, Nicholson P, Doonan JH, Vain P. T-DNA mutagenesis in *Brachypodium distachyon*. Journal of Experimental Botany. 2012; 63: 567–576. doi: 10.1093/jxb/err333 22090444

[31] Olasupo FO, Ilori CO, Forster BP, Bado S. Mutagenic effects of gamma radiation on eight accessions of cowpea (*Vigna unguiculata* [L.] Walp.). American Journal of Plant Sciences. 2016; 7: 339–35. doi: 10.4236/ajps.2016.72034

[32] Brock RD. Prospects and perspectives in mutation breeding. Genetic Diversity in Plants. Basic Life Sciences. 1977; 8: 117–132. doi: 10.1007/978-1-4684-2886-5_12 1073206

[33] Forster BP, Shu QY. Plant mutagenesis in crop improvement: basic terms and applications. In: Shu QY, Forster BP, Nakagawa H, editors. Plant mutation breeding and biotechnology. 2011. Wallingford: CABI, 9–20.

[34] Wilkie KCB. The Hemicelluloses of Grasses and Cereals. Advances in Carbohydrate Chemistry and Biochemistry. 1979; 36: 215–264. doi: 10.1016/S0065-2318(08)60237-1

[35] Van Soest PJ. Nutritional ecology of the ruminant. 2nd Ed. Comstock, Cornell Univ. Press, Ithaca, NY. 1994. 488 p.

[36] Romero N, Leonard I, Ramírez JL, Córdova A. Rendimiento y calidad de la *Clitoria ternatea* en un suelo arcilloso del estado Falcón, Venezuela. Revista Electrónica de Veterinaria. 2013; 14: 1–10.

[37] Bezabih M, Pellikaan WF, Tolera A, Khan NA, Hendriks WH. Chemical composition and in vitro total gas and methane production of forage species from the Mid Rift Valley grasslands of Ethiopia. Grass and Forage Science. 2013; 69: 635–643. doi: 10.1111/gfs.12091

[38] Keys JE Jr, Van Soest PJ, Young EP. Comparative Study of the Digestibility of Forage Cellulose and Hemicellulose in Ruminants and Nonruminants. Journal of Animal Science. 1969; 29: 11–15. doi: 10.2527/jas1969.29111x 5356690

[39] Ramírez S, Domínguez D, Salmerón JJ, Villalobos G, Ortega JA. Contreo en surco y etapa de madurez sobre la producción y calidad del forraje de variedades de avena. Archivos de Zootecnia. 2015; 64: 237–244. doi: 10.21071/az.v64i247.405

[40] Ramírez RG, González-Rodríguez H, Morales-Rodríguez R, Cerrillo-Soto A, Juárez-Reyes A, García-Dessommes GJ, et al. Chemical Composition and Dr y Matter Digestion of Some Native and Cultivated Grasses in Mexico. Czech Journal of Animal Science. 2009; 54: 150–162

[41] González-García H, Sánchez-Maldonado A, Sánchez-Muñoz AJ, Orozco-Erives A, Castillo-Castillo Y, Martínez-De la Rosa R, et al. Valor nutritivo del zacate rosado (*Melinis repens*) y del zacate africano (*Eragrostis lehmanniana*) en Chihuahua. Ciencia en la Frontera. 2017; 14: 7–14.

[42] Njau F, Lwelamira J, Hyandye C. Ruminant livestock production and quality of pastures in the communal grazing land of semi-arid central Tanzania. Livestock Research for Rural Development. 2013; 25: 1–13.

[43] Souza L, Velini ED, Maimoni-Rodella RCS, Martins D. Teores de macro e micronutrientes e a relação c/n de várias espécies de plantas daninhas. Planta Daninha. 1999; 17: 163–167. doi: 10.1590/S0100-83581999000100015

[44] Gutiérrez GOG, Morales NCR, Villalobos JC, Ruíz O, Ortega JA, Palacio J. Composición botánica y valor nutritivo de la dieta consumida por bovinos en un área invadida por pasto rosado [*Melinis repens* (willd.) Zizka]. Revista Mexicana de Ciencias Pecuarias. 2019; 10: 212–226. doi: 10.22319/rmcp.v10i1.4451

[45] Morales NCR, Madrid L, Melgoza A, Martínez M, Arévalo S, Rascón, et al. Análisis morfológico de la diversidad del pasto navajita [*Bouteloua gracilis* (Willd. ex Kunth) Lag. ex Steud.], en Chihuahua, México. Técnica pecuaria en México. 2009; 47: 245–256.

[46] Beltrán LS, García CA, Hernández JA, Loredo C, Urrutia J, González LA, et al. “Navajita Cecilia” *Bouteloua gracilis* H.B.K (Lag.). nueva variedad de pasto para zonas áridas y semiáridas. Revista Mexicana de Ciencias Pecuarias. 2010; 1: 127–130.

[47] Beltrán LS, García CA, Hernández JA, Loredo C, Urrutia J, González LA, et al. “Banderilla Diana” *Bouteloua curtipendula* (Michx.) Torr., nueva variedad de pasto para zonas áridas y semiáridas. Revista Mexicana de Ciencias Pecuarias. 2013; 4: 217–221.

[48] O’Reagain PJ, Mentis MT. The effect of plant structure on the acceptability of different grass species to cattle. Journal of the Grassland Society of Southern Africa. 1989; 6: 163–170. doi: 10.1080/02566702.1989.9648180

[49] Morela F, González V, Castro L. Efecto de la radiación Gamma sobre la diferenciación de plantas de caña de azúcar a partir de callos. Agronomía Tropical. 2002; 52: 311–323.

[50] Khalil SA, Zamir R, Ahmad N. Effect of different propagation techniques and gamma irradiation on major steviol glycoside’s content in *Stevia rebaudiana*. The Journal of Animal & Plant Sciences. 2014; 24: 1743–1751.

