# Supplementary material for: Gamma radiation on seeds of natal grass [Melinis repens (Willd.) Zizka] induced plant’s morphological and nutritional variability
Source: PLoS One. 2022 Jul 29;17(7):e0270935. doi: 10.1371/journal.pone.0270935 (PMC9337687; doi:10.1371/journal.pone.0270935)
Supplement: S1 Appendix — (DOCX) [file pone.0270935.s001.docx]

**S1 APPENDIX.**

**Table A1. Descriptive statistics of 18 morphological variables measured on the evaluated genotypes.**

| Variable | N | Mean | Std Dev | Sum Sq | Min V | Max V | CV (%) |
| --- | --- | --- | --- | --- | --- | --- | --- |
| GH | 75 | 69.33 | 7.37 | 5200 | 45.00 | 80.00 | 10.63 |
| FH | 75 | 45.15 | 4.47 | 3386 | 36.00 | 52.80 | 9.90 |
| PH | 75 | 93.63 | 8.22 | 7022 | 75.00 | 115.00 | 8.78 |
| FPr | 75 | 0.48 | 0.04 | 36.3 | 0.38 | 0.57 | 9.17 |
| TDI | 75 | 1.66 | 0.20 | 124.4 | 1.20 | 1.90 | 12.26 |
| TDE | 75 | 48.17 | 10.83 | 3613 | 24.00 | 79.00 | 22.48 |
| LBL | 75 | 11.18 | 1.01 | 838.41 | 8.97 | 13.77 | 9.04 |
| LBW | 75 | 6.75 | 0.52 | 506.06 | 5.67 | 8.23 | 7.77 |
| BLFL | 75 | 5.11 | 0.69 | 383.5 | 3.60 | 6.50 | 13.58 |
| BWFL | 75 | 4.01 | 0.54 | 301.1 | 3.00 | 5.00 | 13.37 |
| CCI | 75 | 6.79 | 2.10 | 508.96 | 3.75 | 12.62 | 31.00 |
| PL | 75 | 17.08 | 1.73 | 1281 | 11.50 | 20.50 | 10.13 |
| TW | 75 | 11.80 | 3.13 | 885.13 | 5.79 | 26.62 | 26.50 |
| LW | 75 | 10.01 | 1.63 | 750.4 | 7.07 | 14.89 | 16.25 |
| LTr | 75 | 0.90 | 0.27 | 67.48 | 0.40 | 2.14 | 29.94 |
| SW | 75 | 2.32 | 0.95 | 174.07 | 0.60 | 6.05 | 40.73 |
| FW | 75 | 24.13 | 4.58 | 1810 | 15.36 | 43.21 | 18.98 |
| FSr | 75 | 0.09 | 0.02 | 6.98 | 0.04 | 0.14 | 25.48 |

N = evaluated plants, Std Dev. Standard deviation, Sum Sq = sum of squares, Min V = minimum value, Max V = maximum value, CV = coefficient of variation. GH = growth habit (°); FH = foliage height (cm); PH = plant height (cm), FPr = foliage height-plant height ratio (unitless), TDI = tiller diameter (mm), TDE = tillering density (#), LBL = leaf blade length (cm), LBW = leaf blade width (mm), BLFL = blade length of flag leaf (cm), BWFL = blade width of flag leaf (mm), CCI = chlorophyll concentration index, PL = panicle length (cm), TW = tillering weight (g), LW = leaves weight (g), LTr = leaf weight-tillering weight ratio (unitless), SW = seed weight (g), FW = foliage weight without seed (g), and FSr = foliage weight-seed weight ratio (unitless).
